# Chaos in heterogeneous neural networks: II. Multiple activity modes

**DOI:** 10.1186/1471-2202-15-S1-O21

**Published:** 2014-07-21

**Authors:** Merav Stern, Johnatan Aljadeff, Tatyana O Sharpee

**Affiliations:** 1 Dept. of Neuroscience, Columbia University, New York, NY, 10027, USA; The Edmond and Lily Safra Center for Brain Sciences, Hebrew University. Jerusalem, 91905, Israel; 2 Dept. of Physics and Center for Theoretical Biological Physics, University of California San Diego; Computational Neurobiology Laboratory, The Salk Institute for Biological Studies, La Jolla, CA, 92037, USA

## 

We study the activity of a recurrent neural network consisting of multiple cell groups through the structure of its correlations by showing how the rules that govern the strengths of connections between the different cell groups shape the average autocorrelation found in each group. We derive an analytical expression for the number of independent autocorrelation modes the network can concurrently sustain. Each mode corresponds to a non-zero component of the network’s autocorrelation, when it is projected on a specific set of basis vectors. In a companion abstract we derive a formula for the first mode, and hence the entire network, to become active. When the network is just above the critical point where it becomes active all groups of cells have the same autocorrelation function up to a constant multiplicative factor. We derive here a formula for this multiplicative factor which is in fact the ratio of the average firing rate of each group. As the effective synaptic gain grows a second activity mode appears, the autocorrelation functions of each group have different shapes, and the network becomes doubly chaotic. We generalize this result to understand how many modes of activity can be found in a heterogeneous network based on its connectivity structure. Finally, we use our theory to understand the dynamics of a clustered network where cells from the same group are strongly connected compared to cells from different groups. We show how this structure can lead to a one or more activity modes and interesting switching effects in the identity of the dominant cluster.

To model the heteregenous network we include *N* neurons that are divided into *D* groups. The synaptic weight between neurons *i*,*j* is drawn from a centered distribution with standard deviations summarized in a *D*×*D* rule matrix *N^-1/2^G_c_*_(_*_i_*_)_*_d_*_(_*_j_*_)_where *c*(*i*) is the group neuron *i* belongs to. The network obeys the standard rate dynamics (*d/dt*)*x_i_*=*- x_i_*+*∑_j_*_=_*_1...N_J_ij_* tanh*x_j_*. The global behavior of the network changes according to the real part of the eigenvalues of a *D*×*D* matrix *M* whose *c*,*d* element is

**Figure 1:**
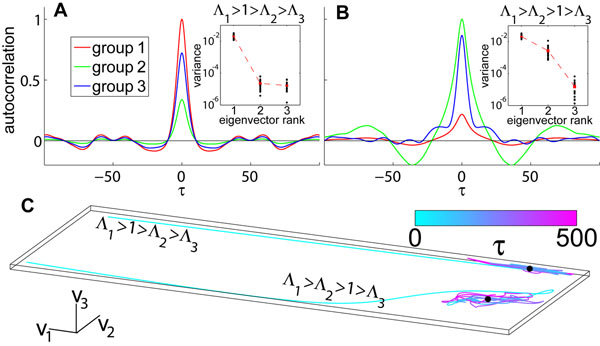
**(A)** For an example network with *1200* neurons divided to *3* equally sized groups we plot the autocorrelation function averaged over neurons belonging to the same group. *G* was chosen such that one eigenvalue of *M* is greater than 1. Independent of the time lag *τ* the autocorrelations maintain a constant ratio that is equal to the ratio of the components of the eigenvector of *M* corresponding to the leading eigenvalue. Inset: for 20 example networks we computed the variance of the autocorrelation vector along the three eigenvectors of *M* and found that the variation in autocorrelation along the leading eigenvector is three orders of magnitude larger than along the other two directions. **(B)** In this example network *M* has two eigenvalues greater than 1. The autocorrelations are no longer a constant ratio of each other, indicating that the network maintains two modes of autocorrelation concurrently. Inset: when averaged over 20 networks we see that the variation along the two eigenvectors with eigenvalues greater than 1 is significantly larger than along the third eigenvector. **(C)** For the two examples networks shown in (A,B) we plotted the trajectory of the autocorrelation vector as a function of the time lag *τ*, and show that they are confined to in the subspace spanned by the eigenvectors, *V_1_ and V_2_* which has eigenvalues greater than 1.

*M_cd_*= *N^-1^N_c_*(*G_cd_*)*^2^*. When *M*'s largest eigenvalue, *Λ_1_*, become larger than 1 the network become chaotic. The ratios of the components of the leading eigenvector *V_1_*are the ratios of the autocorrelations functions of the different groups (Figure 1 A). When *Λ_2_* becomes larger than 1 the network is doubly chaotic (Figure 1 B). In general, the autocorrelation vector has a non-zero projection only on eigenvectors of *M* with eigenvalues greater than 1 (Figure 1 C) and hence the number of active modes in the network is equal to the number of eigenvalues of *M* that are larger than 1.

